# *catena*-Poly[[[diaqua­nickel(II)]-di-μ-glycine] dichloride]

**DOI:** 10.1107/S1600536808015894

**Published:** 2008-06-07

**Authors:** Cynn Dee Ch’ng, Siang Guan Teoh, Suchada Chantrapromma, Hoong-Kun Fun, Siu Mun Goh

**Affiliations:** a School of Chemical Science, Universiti Sains Malaysia, 11800 USM, Penang, Malaysia; b Department of Chemistry, Faculty of Science, Prince of Songkla University, Hat-Yai, Songkhla 90112, Thailand; c X-ray Crystallography Unit, School of Physics, Universiti Sains Malaysia, 11800 USM, Penang, Malaysia

## Abstract

In the polymeric title complex, {[Ni(C_2_H_5_NO_2_)_2_(H_2_O)_2_]Cl_2_}_*n*_, the Ni^II^ atom lies on an inversion center and is in a distorted octa­hedral NiO_6_ configuration, with four carboxyl­ate O atoms from four zwitterionic glycine mol­ecules forming the equatorial plane and two water O atoms occupying the axial positions. The Cl^−^ counterions lie in the inter­stices. The Ni^II^ complexes are linked into polymeric sheets parallel to the *bc* plane. These sheets are then further connected into a three-dimensional network by O—H⋯O, O—H⋯Cl and N—H⋯Cl hydrogen bonds, together with weak C—H⋯O inter­actions.

## Related literature

For values of bond lengths and angles, see: Allen *et al.* (1987 ▶); Shannon (1976 ▶). For related structures, see, for example: Fleck & Bohatý (2005 ▶). For background to the application of nickel complexes, see, for example: Ferrari *et al.* (2002 ▶); Kasuga *et al.* (2001 ▶); Lancaster (1998 ▶); Matkar *et al.* (2006 ▶); Liang *et al.* (2004 ▶).
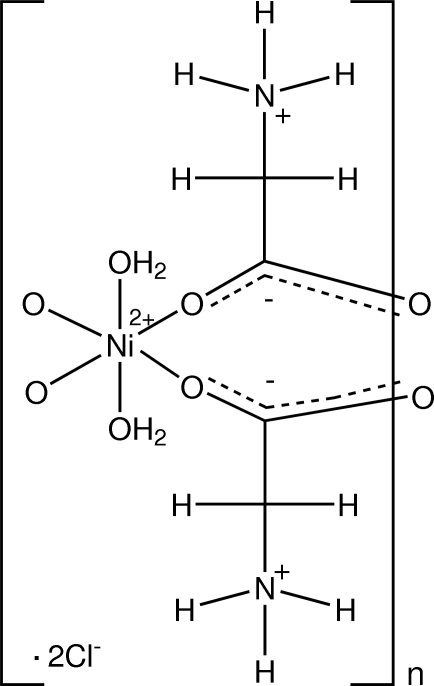


## Experimental

### 

#### Crystal data

[Ni(C_2_H_5_NO_2_)_2_(H_2_O)_2_]Cl_2_
*M* *_r_* = 315.76
Monoclinic, 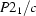

*a* = 10.6006 (1) Å
*b* = 5.8579 (1) Å
*c* = 8.7113 (1) Å
β = 90.489 (1)°
*V* = 540.93 (1) Å^3^
*Z* = 2
Mo *K*α radiation
μ = 2.30 mm^−1^
*T* = 100.0 (1) K
0.32 × 0.22 × 0.12 mm

#### Data collection

Bruker SMART APEX2 CCD area-detector diffractometer
Absorption correction: multi-scan (*SADABS*; Bruker, 2005 ▶) *T*_min_ = 0.530, *T*_max_ = 0.775
11049 measured reflections
2372 independent reflections
2079 reflections with *I* > 2σ(*I*)
*R*_int_ = 0.032

#### Refinement

*R*[*F* ^2^ > 2σ(*F* ^2^)] = 0.023
*wR*(*F* ^2^) = 0.056
*S* = 1.06
2372 reflections
98 parameters
All H-atom parameters refined
Δρ_max_ = 0.49 e Å^−3^
Δρ_min_ = −0.68 e Å^−3^

### 

Data collection: *APEX2* (Bruker, 2005 ▶); cell refinement: *APEX2*; data reduction: *SAINT* (Bruker, 2005 ▶); program(s) used to solve structure: *SHELXTL* (Sheldrick, 2008 ▶); program(s) used to refine structure: *SHELXTL*; molecular graphics: *SHELXTL*; software used to prepare material for publication: *SHELXTL* and *PLATON* (Spek, 2003 ▶).

## Supplementary Material

Crystal structure: contains datablocks global, I. DOI: 10.1107/S1600536808015894/sj2507sup1.cif

Structure factors: contains datablocks I. DOI: 10.1107/S1600536808015894/sj2507Isup2.hkl

Additional supplementary materials:  crystallographic information; 3D view; checkCIF report

## Figures and Tables

**Table 1:** Hydrogen-bond geometry (Å, °)

*D*—H⋯*A*	*D*—H	H⋯*A*	*D*⋯*A*	*D*—H⋯*A*
N1—H1N1⋯Cl1^i^	0.886 (18)	2.326 (17)	3.2021 (9)	170.2 (15)
N1—H2N1⋯Cl1	0.893 (17)	2.404 (17)	3.2673 (11)	162.7 (14)
N1—H3N1⋯Cl1^ii^	0.884 (18)	2.446 (18)	3.2442 (11)	150.4 (15)
O1*W*—H1*W*1⋯O2^iii^	0.840 (18)	2.00 (2)	2.7276 (11)	145 (2)
O1*W*—H2*W*1⋯Cl1	0.81 (2)	2.34 (2)	3.1468 (9)	172.8 (17)
C2—H2*B*⋯O1^iv^	0.938 (17)	2.472 (17)	2.9549 (13)	112.0 (13)

Symmetry codes: (i) 
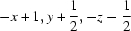
; (ii) 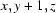; (iii) 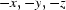; (iv) 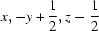.

**Fig. 1.**
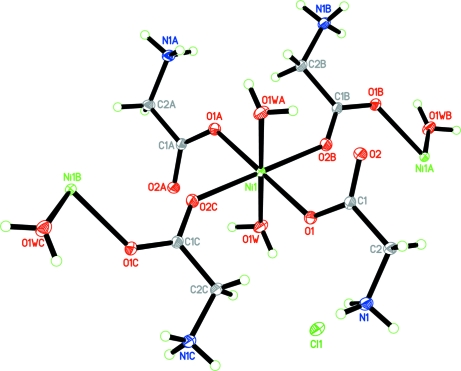
The structure of (I), showing 50% probability displacement ellipsoids and the atomic numbering. Symmetry codes for the (A) -x, -1/2 + y, -1/2 - z, (B) -x, 1/2 + y, -1/2 - z and (C) -x, -y, -z.

**Fig. 2.**
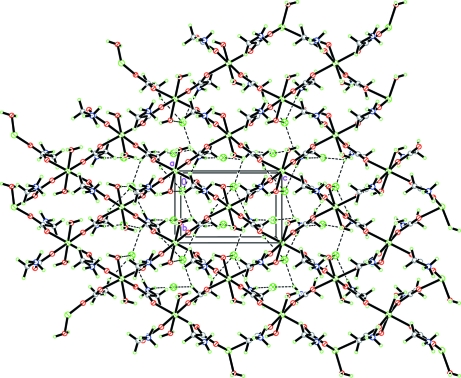
The crystal packing of (I), viewed along the a axis showing the polymeric structure. Hydrogen bonds are drawn as dashed lines.

**Fig. 3.**
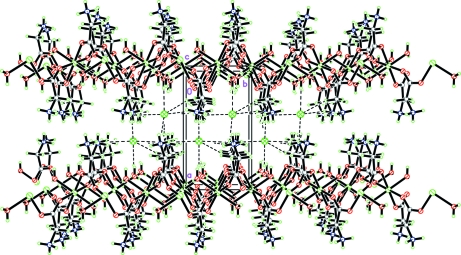
The crystal packing of (I), viewed along the c axis showing the sheets running along the [010] direction. Hydrogen bonds are drawn as dashed lines.

## supplementary crystallographic information

### Comment

Nickel plays versatile and sometimes controversial roles in living systems as the biological effects of nickel are closely related to their chemical nature (Lancaster, 1998). Nickel complexes have been the subject of intense study in recent years mostly due to their biological significance such as antitumor and antibacterial activities (Matkar *et al.*, 2006, Kasuga *et al.*, 2001). Several nickel complexes have been found to inhibit proliferation of diverse cancer cells (Ferrari *et al.*, 2002; Liang *et al.*, 2004, Matkar *et al.*, 2006). Based on the significant biological role played by nickel complexes, we have synthesized several nickel complexes and herein, we report the preparation and crystal structure of the title complex which is isomorphous with *catena*-poly[[[diaquanickel(II)]-di-*µ*- glycine] dibromide] (Fleck & Bohatý, 2005).

In the molecular structure of the polymeric title complex, {[Ni(C_2_H_5_NO_2_)_2_(H_2_O)_2_]Cl_2_}_n_ (Fig. 1), the Ni^II^ lies on an inversion center and has an NiO_6_ coordination environment. The coordination sphere of the Ni^II^ ion is a slightly distorted octahedron consisting of the O_4_ coordination plane of the four glycine zwitterions (coordinating through one carboxylic O atom from each glycine zwitterion) and the two axially bound water molecules. The Ni—O(glycine) distances [Ni1—O1 = 2.0398 (7) Å and Ni1—O2 = 2.0753 (7) Å] and Ni—O(water) distances [2.0413 (8) Å] are quite similar to those observed in another closely related Ni^II^ complex which are in the range 2.033 (2)–2.086 (2) Å (Fleck & Bohatý, 2005) and are also similar to the Ni—O distances observed in ionic compounds (Shannon, 1976). Other bond lengths and angles observed in the structure are also normal (Allen *et al.*, 1987). In the glycine zwitterion, the carboxylate group is slightly twisted from the C1/C2/N1 plane with torsion angles O2—C1—C2—N1 = 167.23 (9)° and O1—C1—C2—N1 - 14.75 (14)°. The C—O distances [C1—O1 = 1.2601 (12) Å and C1—O2 = 1.2524 (12) Å] show some electron delocalization over the carboxylate group. The Cl^-^ ions lie in the interstices between the glycine zwitterions.

The crystal packing in Fig. 2 has shown the polymeric structure of the title polymeric complex. The Ni^II^ complex molecules are linked by O—H···O (Table 1) into polymeric sheets along the [010] direction (Fig. 3). These sheets are furthered connect to the interstial Cl^-^ ions by O—H···Cl and N—H···Cl hydrogen bonds to the water molecules and amino groups, respectively forming a three-dimensional network (Table 1). The crystal is stabilized by O—H···O, O—H···Cl and N—H···Cl hydrogen bonds, together with weak C—H···Cl interactions (Table 1).

### Experimental

The title complex was synthesized by heating under reflux a 1:2 molar mixture of nickel(II) chloride hexahydrate, NiCl_2_.6H_2_O (0.2377 g, 1 mmol) and glycine (0.1503 g, 2 mmol) in water (30 ml) for 3 h. A green transparent solution was obtained and allowed to cool to room temperature. Green single crystals of the title complex suitable for X-ray structure determination were obtained after a few days of evaporation. Mp. 442–443 K.

### Refinement

H atoms were located in difference maps and refined isotropically. The highest residual electron density peak is located at 1.74 Å from O1W and the deepest hole is located at 0.72 Å from Ni1.

### Crystal data

**Table:**

[Ni(C_2_H_5_NO_2_)_2_(H_2_O)_2_]Cl_2_	*F*_000_ = 324
*M**_r_* = 315.76	*D*_x_ = 1.939 Mg m^−^^3^
Monoclinic, *P*2_1_/*c*	Melting point = 442–443 K
Hall symbol: -P 2ybc	Mo *K*α radiation λ = 0.71073 Å
*a* = 10.6006 (1) Å	Cell parameters from 2372 reflections
*b* = 5.8579 (1) Å	θ = 3.8–34.9º
*c* = 8.7113 (1) Å	µ = 2.30 mm^−^^1^
β = 90.489 (1)º	*T* = 100.0 (1) K
*V* = 540.928 (12) Å^3^	Block, green
*Z* = 2	0.32 × 0.22 × 0.12 mm

### Data collection

**Table:**

Bruker SMART APEX2 CCD area-detector diffractometer	2372 independent reflections
Radiation source: fine-focus sealed tube	2079 reflections with *I* > 2σ(*I*)
Monochromator: graphite	*R*_int_ = 0.032
Detector resolution: 8.33 pixels mm^-1^	θ_max_ = 35.0º
*T* = 100.0(1) K	θ_min_ = 3.8º
ω scans	*h* = −17→17
Absorption correction: multi-scan(SADABS; Bruker, 2005)	*k* = −8→9
*T*_min_ = 0.530, *T*_max_ = 0.775	*l* = −14→14
11049 measured reflections	

### Refinement

**Table:**

Refinement on *F*^2^	Secondary atom site location: difference Fourier map
Least-squares matrix: full	Hydrogen site location: inferred from neighbouring sites
*R*[*F*^2^ > 2σ(*F*^2^)] = 0.023	All H-atom parameters refined
*wR*(*F*^2^) = 0.056	*w* = 1/[σ^2^(*F*_o_^2^) + (0.0259*P*)^2^ + 0.1363*P*] where *P* = (*F*_o_^2^ + 2*F*_c_^2^)/3
*S* = 1.06	(Δ/σ)_max_ = 0.001
2372 reflections	Δρ_max_ = 0.49 e Å^−^^3^
98 parameters	Δρ_min_ = −0.68 e Å^−^^3^
Primary atom site location: structure-invariant direct methods	Extinction correction: none

### Special details

**Table:**

Experimental. The low-temperature data was collected with the Oxford Cyrosystem Cobra low-temperature attachment.
Geometry. All e.s.d.'s (except the e.s.d. in the dihedral angle between two l.s. planes) are estimated using the full covariance matrix. The cell e.s.d.'s are taken into account individually in the estimation of e.s.d.'s in distances, angles and torsion angles; correlations between e.s.d.'s in cell parameters are only used when they are defined by crystal symmetry. An approximate (isotropic) treatment of cell e.s.d.'s is used for estimating e.s.d.'s involving l.s. planes.
Refinement. Refinement of *F*^2^ against ALL reflections. The weighted *R*-factor *wR* and goodness of fit *S* are based on *F*^2^, conventional *R*-factors *R* are based on *F*, with *F* set to zero for negative *F*^2^. The threshold expression of *F*^2^ > σ(*F*^2^) is used only for calculating *R*-factors(gt) *etc*. and is not relevant to the choice of reflections for refinement. *R*-factors based on *F*^2^ are statistically about twice as large as those based on *F*, and *R*- factors based on ALL data will be even larger.

### Fractional atomic coordinates and isotropic or equivalent isotropic displacement parameters (Å^2^)

**Table:**

	*x*	*y*	*z*	*U*_iso_*/*U*_eq_	
Ni1	0.0000	0.0000	0.0000	0.00637 (5)	
Cl1	0.38948 (2)	−0.28198 (5)	−0.05133 (3)	0.01159 (6)	
O1	0.14915 (7)	0.13388 (13)	−0.11588 (8)	0.00907 (13)	
O2	0.04973 (7)	0.34293 (14)	−0.29470 (8)	0.01009 (14)	
N1	0.37953 (8)	0.22021 (18)	−0.21419 (11)	0.00996 (16)	
C1	0.14619 (9)	0.25515 (18)	−0.23533 (11)	0.00790 (16)	
C2	0.27217 (9)	0.2880 (2)	−0.31479 (12)	0.01036 (18)	
O1W	0.10514 (8)	−0.28293 (15)	0.04954 (9)	0.01166 (15)	
H2A	0.2839 (16)	0.444 (3)	−0.345 (2)	0.017 (4)*	
H2B	0.2718 (16)	0.195 (3)	−0.4024 (19)	0.018 (4)*	
H1N1	0.4466 (17)	0.204 (3)	−0.2731 (19)	0.020 (4)*	
H2N1	0.3641 (15)	0.088 (3)	−0.1668 (19)	0.015 (4)*	
H3N1	0.3947 (17)	0.323 (3)	−0.142 (2)	0.025 (5)*	
H1W1	0.087 (2)	−0.324 (4)	0.139 (2)	0.038 (6)*	
H2W1	0.180 (2)	−0.284 (3)	0.032 (2)	0.032 (5)*	

### Atomic displacement parameters (Å^2^)

**Table:**

	*U*^11^	*U*^22^	*U*^33^	*U*^12^	*U*^13^	*U*^23^
Ni1	0.00560 (8)	0.00757 (9)	0.00594 (8)	−0.00004 (6)	0.00067 (5)	0.00022 (6)
Cl1	0.00941 (10)	0.01139 (12)	0.01402 (11)	0.00140 (8)	0.00280 (8)	0.00077 (8)
O1	0.0083 (3)	0.0107 (4)	0.0082 (3)	−0.0006 (3)	0.0007 (2)	0.0023 (2)
O2	0.0085 (3)	0.0132 (4)	0.0086 (3)	0.0018 (3)	0.0000 (2)	0.0022 (3)
N1	0.0075 (3)	0.0121 (4)	0.0103 (4)	−0.0001 (3)	0.0009 (3)	0.0021 (3)
C1	0.0079 (4)	0.0085 (4)	0.0074 (4)	−0.0009 (3)	0.0011 (3)	−0.0007 (3)
C2	0.0071 (4)	0.0145 (5)	0.0095 (4)	0.0000 (3)	0.0007 (3)	0.0033 (3)
O1W	0.0089 (3)	0.0129 (4)	0.0133 (3)	0.0023 (3)	0.0028 (3)	0.0027 (3)

### Geometric parameters (Å, °)

**Table:**

Ni1—O1^i^	2.0398 (7)	N1—C2	1.4845 (14)
Ni1—O1	2.0399 (7)	N1—H1N1	0.885 (18)
Ni1—O1W^i^	2.0413 (8)	N1—H2N1	0.893 (18)
Ni1—O1W	2.0414 (8)	N1—H3N1	0.884 (19)
Ni1—O2^ii^	2.0753 (7)	C1—C2	1.5217 (14)
Ni1—O2^iii^	2.0753 (7)	C2—H2A	0.959 (18)
O1—C1	1.2601 (12)	C2—H2B	0.939 (17)
O2—C1	1.2524 (12)	O1W—H1W1	0.84 (2)
O2—Ni1^iv^	2.0753 (7)	O1W—H2W1	0.81 (2)
			
O1^i^—Ni1—O1	180.0	C2—N1—H2N1	111.2 (11)
O1^i^—Ni1—O1W^i^	89.58 (3)	H1N1—N1—H2N1	109.0 (15)
O1—Ni1—O1W^i^	90.42 (3)	C2—N1—H3N1	111.7 (12)
O1^i^—Ni1—O1W	90.42 (3)	H1N1—N1—H3N1	110.1 (16)
O1—Ni1—O1W	89.58 (3)	H2N1—N1—H3N1	107.3 (16)
O1W^i^—Ni1—O1W	180.0	O2—C1—O1	125.94 (9)
O1^i^—Ni1—O2^ii^	86.34 (3)	O2—C1—C2	118.48 (9)
O1—Ni1—O2^ii^	93.66 (3)	O1—C1—C2	115.54 (9)
O1W^i^—Ni1—O2^ii^	87.50 (3)	N1—C2—C1	111.66 (8)
O1W—Ni1—O2^ii^	92.50 (3)	N1—C2—H2A	108.4 (10)
O1^i^—Ni1—O2^iii^	93.66 (3)	C1—C2—H2A	111.2 (10)
O1—Ni1—O2^iii^	86.34 (3)	N1—C2—H2B	108.8 (10)
O1W^i^—Ni1—O2^iii^	92.50 (3)	C1—C2—H2B	107.4 (10)
O1W—Ni1—O2^iii^	87.50 (3)	H2A—C2—H2B	109.4 (14)
O2^ii^—Ni1—O2^iii^	180.0	Ni1—O1W—H1W1	107.7 (15)
C1—O1—Ni1	127.70 (7)	Ni1—O1W—H2W1	120.0 (14)
C1—O2—Ni1^iv^	137.59 (7)	H1W1—O1W—H2W1	114 (2)
C2—N1—H1N1	107.6 (11)		
			
O1W^i^—Ni1—O1—C1	−35.21 (9)	Ni1^iv^—O2—C1—C2	10.54 (16)
O1W—Ni1—O1—C1	144.79 (9)	Ni1—O1—C1—O2	10.33 (16)
O2^ii^—Ni1—O1—C1	−122.74 (9)	Ni1—O1—C1—C2	−167.51 (7)
O2^iii^—Ni1—O1—C1	57.26 (9)	O2—C1—C2—N1	167.23 (9)
Ni1^iv^—O2—C1—O1	−167.25 (8)	O1—C1—C2—N1	−14.75 (14)

Symmetry codes: (i) −*x*, −*y*, −*z*; (ii) *x*, −*y*+1/2, *z*+1/2; (iii) −*x*, *y*−1/2, −*z*−1/2; (iv) −*x*, *y*+1/2, −*z*−1/2.

### Hydrogen-bond geometry (Å, °)

**Table:**

*D*—H···*A*	*D*—H	H···*A*	*D*···*A*	*D*—H···*A*
N1—H1N1···Cl1^v^	0.886 (18)	2.326 (17)	3.2021 (9)	170.2 (15)
N1—H2N1···Cl1	0.893 (17)	2.404 (17)	3.2673 (11)	162.7 (14)
N1—H3N1···Cl1^vi^	0.884 (18)	2.446 (18)	3.2442 (11)	150.4 (15)
O1W—H1W1···O2^vii^	0.840 (18)	2.00 (2)	2.7276 (11)	145 (2)
O1W—H2W1···Cl1	0.81 (2)	2.34 (2)	3.1468 (9)	172.8 (17)
C2—H2B···O1^viii^	0.938 (17)	2.472 (17)	2.9549 (13)	112.0 (13)

Symmetry codes: (v) −*x*+1, *y*+1/2, −*z*−1/2;  (vi) *x*, *y*+1, *z*; (vii) −*x*, −*y*, −*z*; (viii) *x*, −*y*+1/2, *z*−1/2.
